# Studies on the Anti-Oxidative Function of *trans*-Cinnamaldehyde-Included β-Cyclodextrin Complex

**DOI:** 10.3390/molecules22121868

**Published:** 2017-12-19

**Authors:** Munkhtugs Davaatseren, Yeon-Ji Jo, Geun-Pyo Hong, Haeng Jeon Hur, Sujin Park, Mi-Jung Choi

**Affiliations:** 1 Department of Food Science and Technology, Chung-ang University, Gyeonggi-do 17546, Korea; munkhtugs@hotmail.com; 2 Institute of Process Engineering in Life Science, Section I: Food Process Engineering, Karlsruhe Institute of Technology, 76131 Karlsruhe, Germany; jo.yeonji.1986@gmail.com; 3 Department of Food Science and Biotechnology, Sejong University, 209 Neungdong-ro, Seoul 05006, Korea; 4 Division of Metabolism and Functionality Research, Korea Food Research Institute, 1201-62 Anyangpangyo-ro, Bundang-gu, Seongnam-si, Gyeonggi-do 13539, Korea; mistletoe@kfri.re.kr (H.J.H.); parksj83@naver.com (S.P.); 5 Department of Food Science and Biotechnology of Animal Resources, Konkuk University, 120 Neungdong-ro, Seoul 05029, Korea

**Keywords:** *trans*-cinnamaldehyde, β-cyclodextrin, self-inclusion, anti-inflammation, antioxidant

## Abstract

*trans*-Cinnamaldehyde (*t*CIN), an active compound found in cinnamon, is well known for its antioxidant, anticancer, and anti-inflammatory activities. The β-cyclodextrin (β-CD) oligomer has been used for a variety of applications in nanotechnology, including pharmaceutical and cosmetic applications. Here, we aimed to evaluate the anti-inflammatory and antioxidant effects of *t*CIN self-included in β-CD complexes (CIs) in lipopolysaccharide (LPS)-treated murine RAW 264.7 macrophages. RAW 264.7 macrophages were treated with increasing concentrations of β-CD, *t*CIN, or CIs for different times. β-CD alone did not affect the production of nitric oxide (NO) or reactive oxygen species (ROS). However, both *t*CIN and CI significantly reduced NO and ROS production. Thus, CIs may have strong anti-inflammatory and antioxidant effects, similar to those of *t*CIN when used alone.

## 1. Introduction

Cinnamon is commonly used in cosmetics and foods [1], and cinnamon oil is frequently used in the food and beverage industry because of its unique aroma [2]. Several studies have reported that cinnamon and its extracts and active compounds have beneficial biological effects, including antidiabetic effects [1,3], and antibacterial, antifungal, and anticancer activities [2,4]. Moreover, these products inhibit neuroinflammation [5] and reduce oxidative stress [6,7].

The compound *trans*-cinnamaldehyde (*t*CIN) is a key flavor component of cinnamon essential oil [8] that has relatively low toxicity, aside from inducing skin irritation at high doses [9]. Several reports have suggested that *t*CIN has anti-inflammatory effects in macrophages [10,11]. Moreover, *t*CIN has anticancer activity, induces apoptosis [8], inhibits cell proliferation [12], and is beneficial for the management of obesity and diabetes [13]. However, the application of *t*CIN is limited by its insolubility in water; therefore, overcoming this issue could have a major impact on the functionality of *t*CIN. In addition, microencapsulated *t*CIN has no carcinogenic or toxic effects in rodent models [14]. However, the oral absorption of high amounts of cinnamon into the human body is likely to cause side effects such as hyperkeratosis and gastritis [15]. Therefore, the cytotoxicity of *t*CIN should be carefully examined.

Cyclodextrins (CDs) are toroidal-shaped, biocompatible, relatively non-toxic, cyclic oligomers [16]. In aqueous solutions, CDs can incorporate geometrically and polarity-compatible target compounds to improve their stability [17], increase their solubility [18], and enhance their bioavailability [19]. This increases the applicability of CDs in many fields, including pharmaceutics [16,20], cosmetics [17], and food technology [21,22]. Therefore, many researchers have deemed CDs as potential specific drug carriers or nano-inclusion agents with the ability to reduce the toxicity of target compounds, after numerous modifications [23]. CDs are thought to be suitable for use in several pharmacological and biological approaches, helping to address the challenges faced during product formulation. CD encapsulation usually affects the physicochemical properties of bioactive compounds and specific drugs [17,24]. However, few studies have examined *t*CIN and CD inclusion complexes, and most studies have focused only on their applications in nanotechnology. In particular, researchers are interested in elucidating the functionality of these molecules using cell-based experiments; additionally, more in-depth studies are needed to uncover the potential applications of these compounds. Because *t*CIN is a major component of cinnamon and generally considered as a food, *t*CIN could be applied as a functional food with numerous beneficial effects after modification by nanotechnology. Here, we evaluated the physicochemical properties of the β-CD and *t*CIN inclusion complexes (CIs), and determined their anti-inflammatory and antioxidant effects on lipopolysaccharide (LPS)-treated RAW 264.7 murine macrophages.

## 2. Results and Discussion

### 2.1. Thermal Properties

**Figure 1:**
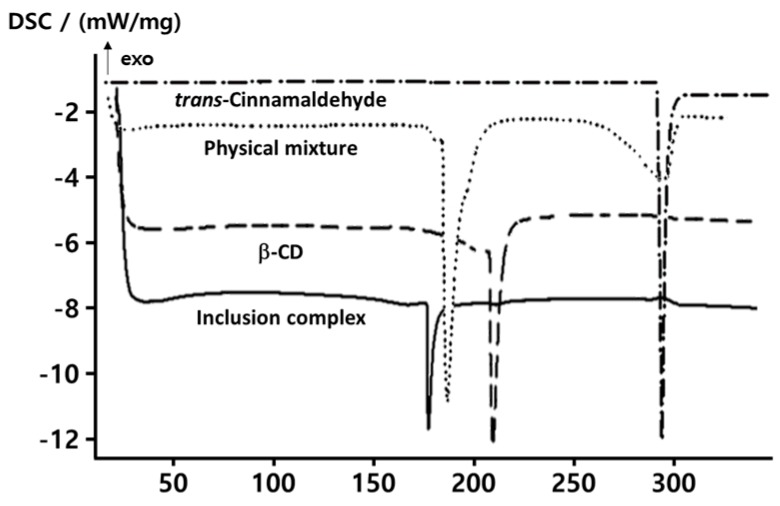
Thermal analysis of *trans*-cinnamaldehyde (*t*CIN) and β-cyclodextrin (β-CD) inclusion complexes (CIs). The CIs were obtained by the molecular inclusion of *t*CIN and β-CD at a molar ratio of 1:1. The physical mixture was obtained by pulverizing the two components in a glass mortar and mixing them accurately in a molar ratio of 1:1.

Differential scanning colorimetric (DSC) was performed to investigate the formation of complexes between the β-CD polymer and *t*CIN. Figure 1 shows the DSC results for β-CD, *t*CIN, the β-CD-*t*CIN physical mixture, and the CIs. Pure β-CD and pure *t*CIN showed endothermic peaks at 205.5 °C and 292.7 °C, respectively, which correspond to their melting points. The β-CD-*t*CIN physical mixture had two endothermic peaks. The first (at 183.7 °C) was nearly identical to that of pure β-CD, while the second (at 292.4 °C) corresponded to that of *t*CIN. The melting temperature of β-CD alone was higher than that of the CIs. Interaction of the guest with β-CD provides somewhat broader, so that a difference in phase transition temperature is observed [25]. The thermal transition of the CIs occurred at 175.8 °C, along with the endothermic peak. The CIs did not show the *t*CIN melting peak, which was clear evidence of the formation of a complex between the β-CD and *t*CIN. Similar to our results, Seo et al. [25] previously reported that the disappearance of the endothermic peak of eugenol was obvious evidence of the formation of an inclusion complex between eugenol and β-CD.

### 2.2. Encapsulation Efficiency and Release Characteristics of the CIs

**Figure 2:**
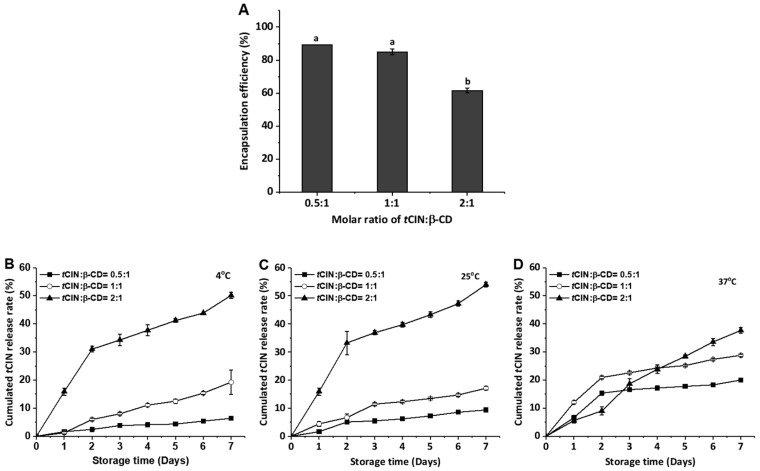
Encapsulation efficiency (**A**) and release amount (**B**–**D**) of the inclusion complex (CI) with different ratios of *trans*-cinnamaldehyde (*t*CIN) and β-cyclodextrin (β-CD). The release amount was measured at different storage temperatures, i.e., 4 °C (**B**), 25 °C (**C**), and 37 °C (**D**), for 7 days and expressed as a percentage (%).

Cinnamon essential oil and *t*CIN have antibacterial and antifungal effects [2], and have been shown to be promising agents in the treatment of cancer [4]. Despite the beneficial effects of cinnamon, its efficacy and bioavailability are quite low because it is used in low doses for oral absorption in the human body [26]. Thus, the encapsulation efficiency of CIs with different molar ratios of β-CD and *t*CIN was measured before evaluating the *t*CIN release rate. The encapsulation efficiency of CI decreased from 90% to 62%, with increasing *t*CIN concentrations in the CIs. The encapsulation efficiency when the molar ratio of the two components was 1:1, was 85% (Figure 2A). Similar observations were reported in previous studies, with encapsulation efficiencies ranging from 70% to 95% after the preparation of CIs with various concentrations of wall and core materials [27]. Hill, Gomes, and Taylor [27] found that the encapsulation efficiency for *t*CIN in β-CD inclusion complexes was 85% when the inclusion complexes were prepared at a molar ratio of 1:1, similar to our current results. Many studies have shown that the type of material used for preparing the wall, the ratio of the materials used for the core and the wall, the encapsulation technique, and the physicochemical properties of capsules affect the encapsulation efficiency value [28,29,30,31]. In particular, the CI technique is effective for encapsulating highly lipophilic oils with high encapsulation efficiency when prepared such that the molar ratio of the materials used for the core and wall is 1:1. In contrast, in our study, at a 1:1 molar ratio of CI and *t*CIN, the CI had the lowest encapsulation efficiency.

The release profiles of *t*CIN from CIs were observed to determine the stability of the CIs at 4, 25, and 37 °C over a period of 7 days (Figure 2B–D). The *t*CIN release rate tended to increase over time as the *t*CIN concentration increased. The CIs at a 0.5:1 molar ratio of *t*CIN and β-CD were more stable than those at a 1:1 and a 2:1 molar ratio. Especially, the release rate of *t*CIN from CI dynamically increased at a 2:1 molar ratio of *t*CIN and β-CD, to up to about 40%, regardless of the temperature.

In general, the storage temperature had a pronounced effect on the release rate of *t*CIN from CIs. At 4 °C and 25 °C, *t*CIN release from CIs did not differ significantly with storage temperature (*p* > 0.05) in our study. However, *t*CIN release from CIs was more affected by storage temperature at 37 °C than at the other storage temperatures, even though the concentration was low. *t*CIN release from CIs at the molar ratios of 0.5:1 and 1:1, and a 2:1 molar ratio of *t*CIN and β-CD, was 20%, 28%, and 37%, respectively. According to a study by Wang et al. [30], the release rate of garlic oil from the inclusion complex, examined at temperatures ranging from 25 to 50 °C, reached 75.8% at 37 °C after incubation for 60 h. Thus, the release profiles of the core materials can be controlled to suit a given application using various types and concentrations of coating materials, different encapsulation techniques, and various extra-environmental conditions, such as temperature, pH, and humidity [32,33]. In this study, functionality evaluation was performed to observe the effects of *t*CIN release on anti-inflammatory and antioxidant activity.

### 2.3. Anti-Oxidant Activities of the CIs

**Figure 3:**
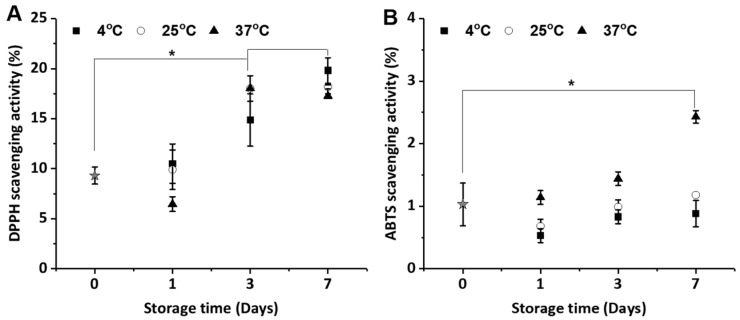
Antioxidant activity of *trans*-cinnamaldehyde (*t*CIN) and β-cyclodextrin (β-CD) inclusion complex (CI). (**A**) DPPH and (**B**) ABTS radical-scavenging activities were measured at different storage temperatures (4 °C, 25 °C and 37 °C) for 7 days. The CIs were obtained by molecular inclusion at 1:1 molar ratio of *t*CIN and β-CD. Data are the mean ± standard deviation (SD). * *p* < 0.05 versus the radical-scavenging ability of the CIs at day 0 (initial CIs). DPPH, 1,1-diphenyl-2-picrylhydrazyl; ABTS, 2,20-azino-bis(3-ethylbenzothiazoline-6-sulphonic) acid.

Figure 3 shows the antioxidant activity of the CIs over a period of 7 days, as measured by the 1,1-diphenyl-2-picrylhydrazyl (DPPH) (Figure 3A) and 2,20-azino-bis(3-ethylbenzothiazoline-6-sulphonic) acod (ABTS) (Figure 3B) radical-scavenging activity assays. These antioxidant activities were investigated to evaluate the storage stability of CI-containing antioxidants, such as *t*CIN*.* In our study, The CIs were obtained by molecular inclusion at 1:1 molar ratio of *t*CIN and β-CD. Free β-CD did not show any antioxidant activity on its own when tested at the same concentration range as carvacrol and its inclusion complexes (data not shown). Free *t*CIN did not show any antioxidant activity because of its instability in distilled water. However, *t*CIN is well-known for its high antioxidant activity in previous studies [6,7]. In the DPPH and ABTS radical-scavenging assay, the antioxidant activity of the CIs significantly increased after 3 days compared with that of the CIs at day 0 (initial CIs). The results of the ABTS assay also showed that the antioxidant activity of the CIs increased with increasing storage temperature and time. In general, the inclusion of *t*CIN with β-CD makes it difficult to react with free radicals. However, *t*CIN is released from CIs, and this free *t*CIN reacts with free radicals, eventually increasing the antioxidant activity [34]. This could be also explained with the results of release rate (%) presented in Figure 2B–D; after 7 days, *t*CIN release from CIs at 4 °C, 25 °C, and 37 °C was up to 19%, 17% and 30%, respectively. The ABTS radical-scavenging activity of CIs at 4 °C, 25 °C, and 37 °C was 0.88%, 1.18%, and 2.43%, respectively. Therefore, we suggest that the increase in the antioxidant activity was most likely related to the free *t*CIN concentration, because an increased release of *t*CIN was observed with increasing storage temperatures.

### 2.4. Cell Viability

**Figure 4:**
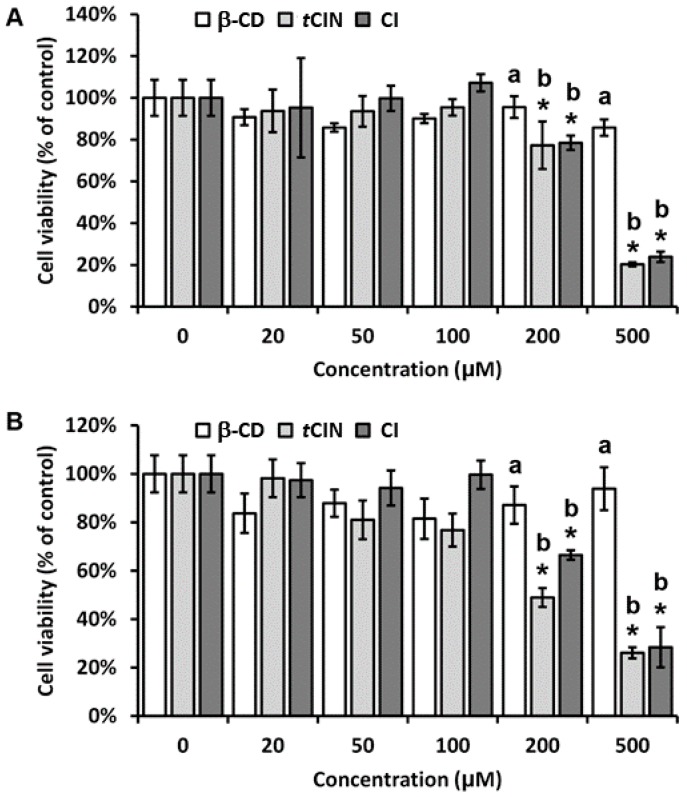
Cell viability. RAW 264.7 macrophages were seeded in 96-well plates for 24 h, followed by 18 h of starvation. Cells were treated with increasing concentrations of β-CD, *t*CIN, and CIs for 24 h. Cell viability was measured using the MTT assay, and quantified as a percentage (%) of the control. MTT assays were performed at (**A**) week 0 and (**B**) after 3 weeks of storage at 4 °C to evaluate the stability of CI and the effects of storage on CI. The CI was obtained by molecular inclusion at a 1:1 molar ratio of *t*CIN and β-CD. Data are the mean ± standard deviation (SD). * *p* < 0.05 versus the control. The different letters indicate *p* < 0.05 at the same treatment concentration. β-CD, β-cyclodextrin; *t*CIN, *trans*-cinnamaldehyde; CI, *t*CIN and β-CD inclusion complexes; MTT, 3-(4,5-dimethylthiazol-2-yl)-2,5-diphenyltetrazolium bromide.

In order to determine whether the storage time of the CIs affected their influence on cell viability, CIs were prepared in a culture medium and stored at 4 °C, and cell viability was determined before and after 3 weeks of storage. According to the MTT assay data, β-CD exhibited no significant cytotoxicity at a concentration of 500 μM, whereas *t*CIN and CI exhibited no significant cytotoxicity up to a concentration of 100 μM in RAW 264.7 macrophages (Figure 4). Interestingly, a 3-week storage (Figure 4B) slightly increased the cytotoxicity of *t*CIN, when compared to that observed before storage (Figure 4A); this could be caused by the oxidation of *t*CIN during storage. However, 3 weeks of storage of the CIs in a culture medium at 4 °C had no notable effect on cell viability. This result supported the findings of a previous report by Yang et al. [35], who demonstrated that the cytotoxicity of a target compound decreased after β-CD inclusion. Therefore, the *t*CIN and β-CD inclusion complex may have an important role in maintaining the stability of functional foods.

### 2.5. Inhibition of NO Production

**Figure 5:**
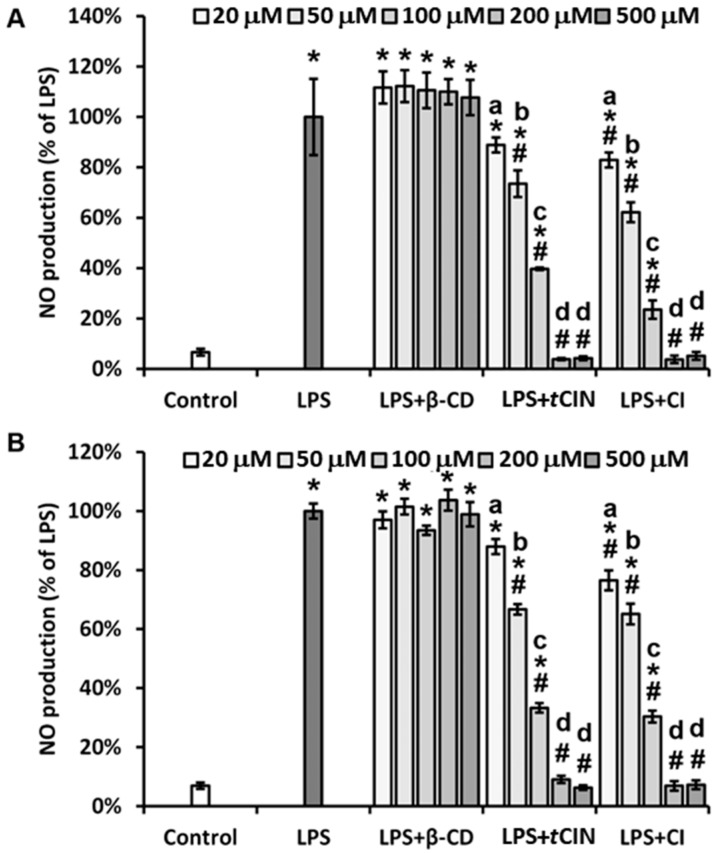
Inhibition of nitric oxide (NO) production by the *trans*-cinnamaldehyde (*t*CIN) and β-cyclodextrin (β-CD) inclusion complex (CI). RAW 264.7 macrophages were seeded in 96-well plates for 24 h, followed by 18 h of starvation. Cells were treated with increasing concentrations of β-CD, tCIN, and CIs for 30 min, and were then treated with LPS for 24 h. NO production was determined using an NO detection kit, according to the manufacturer’s protocol. NO production was determined at (**A**) week 0 and (**B**) after 3 weeks of storage at 4 °C to evaluate the stability and effects of storage on CIs. The CI was obtained by molecular inclusion at a 1:1 molar ratio of *t*CIN and β-CD. Data are the mean ± standard deviation (SD). * *p* < 0.05 versus the control, # *p* < 0.05 versus LPS. The different letters indicate *p* < 0.05 within the same treatment group. LPS, lipopolysaccharide.

LPS, which is a cell wall component of Gram-negative bacteria, activates macrophages and triggers inflammatory responses by producing pro-inflammatory cytokines and mediators. These mediators, including inducible nitric oxide synthase (iNOS), cyclooxygenase (COX)-2, tumor necrosis factor (TNF)-α, and interleukin (IL)-1β, can be released by various cells, including murine RAW 264.7 macrophages. Alternatively, iNOS expression and NO production are known to be beneficial in both acute and chronic inflammation [36]. According to Lee and Choi [29], cinnamon extracts significantly inhibit NO production. Ho et al. [5] reported that among the major components of cinnamon, *t*CIN shows the highest inhibition of NO production and iNOS expression at both the protein and mRNA levels, at concentrations of 25–100 μM. We evaluated the effects of CIs on NO production in LPS-induced RAW264.7 macrophages. The NO production assays were performed to determine whether storage affected the stability of the CIs. The CIs were prepared in a culture medium and stored at 4 °C, and the production of NO was determined before and after 3 weeks of storage. We evaluated whether the storage time affected NO reduction. For this, CIs were prepared in a culture medium and stored at 4 °C, and the production of NO was determined before and after 3 weeks of storage. We found that treatment with β-CD alone had no effect on NO production induced by LPS in RAW macrophages. In contrast, treatment with only *t*CIN significantly reduced NO production. Interestingly, CI treatment had effects similar to that of treatment with *t*CIN alone; it resulted in reduced NO production, even after storage of samples for 3 weeks at 4 °C (Figure 5).

### 2.6. ROS Suppression

**Figure 6:**
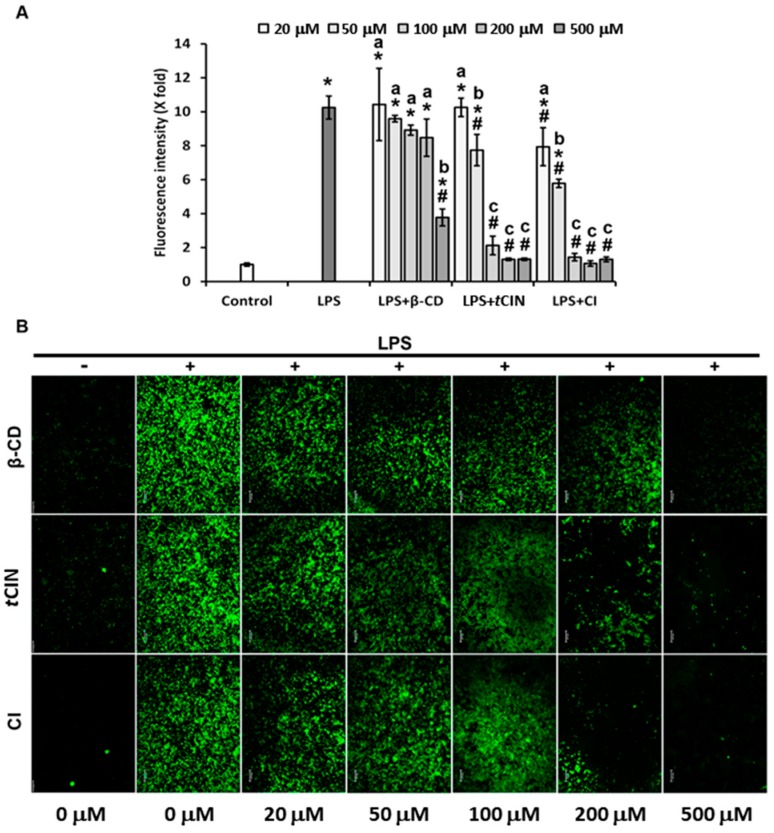
Inhibition of reactive oxygen species (ROS) production by the *trans*-cinnamaldehyde (*t*CIN) and β-cyclodextrin (β-CD) inclusion complexes (CIs). RAW 264.7 macrophages were seeded in 96-well plates with black walls and transparent bottoms for 24 h, and then subjected to starvation for 18 h. Cells were treated with increasing concentrations of β-CD, *t*CIN, and CIs for 30 min, and then treated with LPS for 24 h. ROS levels were determined using H2DCFDA, according to the manufacturer’s protocol. (**A**) The fluorescence intensity was measured using a fluorescent microplate reader, and quantified as fold change compared to that of the control group; (**B**) Intensity was visualized using a fluorescence microscope at 100× magnification. The CIs were obtained by molecular inclusion at a 1:1 molar ratio of *t*CIN and β-CD. Data are the mean ± standard deviation (SD). * *p* < 0.05 versus the control, # *p* < 0.05 versus LPS. The different letters indicate *p* < 0.05 within the same treatment group. LPS, lipopolysaccharide; H2DCFDA, 2′,7′-dichlorodihydrofluorescein diacetate.

To evaluate the antioxidant effects of the CIs, the levels of ROS were determined in LPS-treated RAW 264.7 macrophages using 2′,7′-dichlorodihydrofluorescein diacetate (H2DCFDA, D-399). The LPS-treated cells showed significantly higher levels of fluorescence than the untreated control, and *t*CIN significantly reduced this effect (Figure 6). This result is similar to that reported by Lee et al. [11], wherein *t*CIN was shown to inhibit LPS-induced ROS generation in J774A.1 macrophages. Interestingly, as shown in Figure 6B, *t*CIN self-inclusion in β-CD improved the ROS-reduction effect in LPS-treated RAW 264.7 cells. At *t*CIN and CI concentrations above 100 μM, the LPS-induced ROS level was reduced by approximately 5-fold (Figure 6A), indicating that CIs could be used as potential antioxidant agents. However, additional in vitro and in vivo studies of the effects of CIs on inflammation and oxidative stress are required. In addition, its underlying mechanisms need to be studied further for a better understanding and elucidation of its beneficial effects.

## 3. Materials and Methods

### 3.1. Materials

Murine RAW264.7 macrophages were purchased from the American Type Culture Collection (ATCC TIB-71; ATCC, Manassas, VA, USA). Dulbecco’s modified Eagle’s medium (DMEM; low glucose, 1000 mg/L; phenol red, LM 001-11) and fetal bovine serum (FBS; S 001-07) were purchased from Welgene Inc. (Daegu, Korea). LPS (cat. no. L6529), *t*CIN (cat. no. C80687), vitamin C (cat. no. A0278), 1,1-diphenyl-2-picrylhydrazyl (DPPH; cat. no. D9132), and 2,2′-azino-bis (3-ethylbenzothiazoline-6-sulfonic acid) diammonium salt (ABTS; cat. no. A1888) were purchased from Sigma-Aldrich (St. Louis, MO, USA). β-CD (cat. no. 030-08342) was purchased from Wako Pure Chemical Industries, Ltd. (Osaka, Japan). The nitric oxide (NO) detection kit (cat. no. ADI-917-010) was purchased from Enzo Life Sciences (Farmingdale, NY, USA).

### 3.2. Sample Preparation

For in vitro experiments, *t*CIN, β-CD, and CI samples were prepared in distilled water (for test of encapsulation efficiency, release study, and anti-oxidant activity) or cell culture medium (for test of cell viability, NO production, and ROS determination). 1 mM each of β-CD (pure powder) and *t*CIN (predissolved in dimethyl sulfoxide [DMSO]) were dissolved in distilled water or culture medium using a shaking incubator at 200 rpm. For CI preparation, 1 mM β-CD (pure powder) was dissolved in distilled water or culture medium in a shaking incubator at 200 rpm for 30 min, and *t*CIN was then added to the solution at the molar ratios of 0.5:1, 1:1, 1:2. The mixture was then placed in a shaking incubator at 200 rpm and 55 °C for 6 h for encapsulation by self-assembling aggregation. The CI samples were stored at 4 °C. The physical mixture was obtained by pulverizing the two components in a glass mortar, and mixing accurately weighed (1:1 molar ratio) amounts of *t*CIN and β-CD.

### 3.3. DSC Measurement

Differential scanning colorimetric (DSC) studies were performed using a DSC 200F3 apparatus (Netzsch-Geraetebau GmbH, Selb, Germany) to confirm the formation of the CIs. β-CD, *t*CIN, the β-CD-*t*CIN physical mixture, and CIs were analyzed. The β-CD-*t*CIN physical mixture was prepared. The temperature was calibrated using indium. The samples were weighed with an accuracy of 3 ± 0.01 mg and hermetically sealed in an aluminum pan. Each sample was scanned from 20 to 300 °C, with the heating set at 10 °C/min under nitrogen gas injection.

### 3.4. Encapsulation Efficiency and Release Study

The encapsulation efficiency (EE%) of *t*CIN was determined using a UV/VIS spectrophotometer (OPTIZEN, Mecasys Co., Daejeon, Korea). To extract free *t*CIN, *n*-hexane (9 mL) and the CIs (1 mL) were mixed together and centrifuged at 4000 rpm for 10 min. The extracted free *t*CIN in the supernatant of *n*-hexane was determined using an ultraviolet (UV)/visible (VIS) spectrophotometer at 285 nm. The EE% was indirectly calculated using a calibration curve constructed from the values of a series of *t*CIN solutions in n-hexane with standard concentrations. The EE% was then obtained as a percentage from the following equation:

(1) $$\mathrm{Encapsulation}\;\mathrm{efficiency}\;\left(\%\right)=\;\frac{\mathrm{Total}\;\mathrm{amount}\;\mathrm{of}\;t\mathrm{CIN}\;\left[g\right]-\mathrm{Free}\;\mathrm{amount}\;\mathrm{of}\;t\mathrm{CIN}\;\left[g\right]}{\mathrm{Total}\;\mathrm{amount}\;\mathrm{of}\;t\mathrm{CIN}\;\left[g\right]}$$

The CIs were stored at different temperatures to determine the amount of *t*CIN released. The *t*CIN released from the CIs was determined at intervals using a UV/VIS spectrophotometer, according to the protocol followed by Chun et al. [37], with modifications. The CIs were stored at 4 °C in a refrigerator and at 25 and 37 °C in an incubator. A 1-mL aliquot of the *t*CIN emulsion was withdrawn at week 4, and the amount of *t*CIN extracted was measured as described above. The amount of *t*CIN released was expressed as a percentage of the initial total amount of *t*CIN.

### 3.5. Anti-Oxidative Activity

The antioxidant capacity of the CIs was measured using the DPPH free radical-scavenging and ABTS radical-scavenging capacity methods according to Brand-Williams et al. [38], and Re, et al. [39], respectively. Vitamin C (ascorbic acid, 1 mg/mL) was used as a positive control, and the free radical-scavenging capacity was expressed as a percentage. All determinations were performed at least in triplicate.

### 3.6. Cell Culture

Murine RAW264.7 macrophages were subcultured to 70–80% confluence every 2–3 days in 100-mm dishes (Falcon, Bedford, MA, USA) in DMEM supplemented with 10% FBS, and were incubated in a humidified atmosphere containing 5% CO_2_ and 95% air at 37 °C. For the experiments, the cells were seeded in 96-well plates for cell cytotoxicity, reactive oxygen species (ROS), and nitric oxide (NO) determination in DMEM containing 10% FBS for 24 h. The day before treatments, all cells were starved in DMEM containing 1% FBS overnight, and then treated with β-CD, *t*CIN, or CIs with or without 1 µg/mL LPS for further experiments.

### 3.7. Cell Viability

The protective effects of *t*CIN and β-CD CIs were evaluated in LPS-treated RAW cells using a 3-(4,5-dimethylthiazol-2-yl)-2,5-diphenyltetrazolium bromide (MTT) assay. The β-CD, *t*CIN, and CI samples were prepared and stored at 4 °C for 3 weeks to evaluate the effect of their stability on cell viability. Cell viability experiments were performed using fresh samples and samples stored for 3 weeks. RAW cells (4 × 10^4^ cells/well) were seeded in 96-well plates for 24 h and then starved in DMEM supplemented with 1% FBS overnight before treatment. The cells were treated with different concentrations of β-CD, *t*CIN, and CIs for 24 h, and cell viability was assessed using the MTT assay. The absorbance was measured using an enzyme-linked immunosorbent assay (ELISA) plate reader (Thermo Scientific Multiskan GO microplate spectrophotometer; Thermo Scientific, Lafayette, CO, USA) at 540 nm, and cell viability was determined as a percentage of the control cells.

### 3.8. NO Production

The cells were prepared as described for the MTT assay. RAW264.7 macrophages (4 × 10^4^ cells/well) were seeded in 96-well plates for 24 h and then starved in DMEM supplemented with 1% FBS overnight before treatment. After starvation, the cells were pre-incubated with different concentrations of β-CD, *t*CIN, and CIs for 30 min, and then stimulated with LPS (1 μg/mL). After 24 h, the supernatant was collected, and NO production was determined using an NO detection kit, according to the manufacturer’s protocol.

### 3.9. ROS Determination

The level of intracellular ROS induced by LPS was determined using 2′,7′-dichloro-dihydrofluorescein diacetate (H2DCFDA, D-399), also known as dichlorofluorescin diacetate (Life Technologies Korea LLC, Seoul, Korea), according to the manufacturer’s protocol. Briefly, RAW cells were seeded in black-walled, transparent-bottom 96-well plates (Thermo Scientific Nunc, Rochester, NY, USA) for 24 h and starved overnight before treatment, as described above. The cells were treated with 20 µM H2DCFDA for 1 h in a humidified cell culture incubator and washed twice with phosphate-buffered saline (PBS). H2DCFDA fluorescence was analyzed using a Spectra Max M2e spectrophotometer (Molecular Devices, Bath, UK), at an excitation wavelength of 485 nm, and the fluorescein signal was detected at an emission wavelength of 535 nm. The relative ratio of each sample intensity was calculated as a percentage of the control group value. Fluorescence images were obtained using a Nikon Eclipse Ti fluorescent microscope (Nikon Inc., Tokyo, Japan) at 100× magnification.

### 3.10. Statistical Analysis

Data are presented as the mean ± standard deviation (SD). The significance of differences between groups was assessed using multiple comparisons and analysis of variance (ANOVA), followed by the Tukey honest significant difference (HSD) test. Differences with P values of less than 0.05 were considered statistically significant.

## 4. Conclusions

In this study, *t*CIN was solubilized by formulating it as an inclusion complex with β-CD polymer using molecular inclusion techniques. The encapsulation efficiency was confirmed to be 85%, and high retention of *t*CIN was maintained for 4 weeks. In addition, *t*CIN self-inclusion in the β-CD polymer did not elevate the toxicity to more than that of *t*CIN alone. In fact, the CIs appeared to prevent the oxidation of *t*CIN during prolonged storage. NO assays revealed that the β-CD self-inclusion method did not affect the NO-reducing effects of *t*CIN, even after 3 weeks of storage. Furthermore, the results of DPPH and ABTS radical-scavenging activity assay, and the DCF-DA assay showed that β-CD self-inclusion had no negative effects on the anti-oxidative properties of *t*CIN. Collectively, these results indicated that *t*CIN self-inclusion in β-CD could play an important role in developing nano-functional food applications.
